# Improvement of quantitative solution ^31^P NMR analysis of soil organic P: a study of spin–lattice relaxation responding to paramagnetic ions

**DOI:** 10.1186/s12932-020-00067-7

**Published:** 2020-02-17

**Authors:** Yunbin Jiang, Fengmin Zhang, Chao Ren, Wei Li

**Affiliations:** 1 Key Laboratory of Surficial Geochemistry, Ministry of Education, School of Earth Sciences and Engineering, Nanjing University, Nanjing, 210023 Jiangsu China; 2 Testing Center, Yangzhou University, Yangzhou, 225009 Jiangsu China

**Keywords:** Soil organic P, Solution ^31^P NMR spectroscopy, Spin–lattice relaxation, Paramagnetic ions, Recycle delay time

## Abstract

Solution ^31^P nuclear magnetic resonance (NMR) spectroscopy has been widely applied to analyze the speciation of soil organic P; however, this time-consuming technique suffers from a low analytical efficiency, because of the lack of fundamental information such as the spin–lattice relaxation (*T*_1_) of ^31^P nucleus for model P compounds. In this study, we for the first time determined the *T*_1_ values of twelve typical soil organic P compounds using the inversion recovery method. Furthermore, we examined the effect of co-existing paramagnetic ions (e.g., Fe^3+^ and Mn^2+^) on the reduction of the *T*_1_ values of these compounds. Without the addition of paramagnetic ions, the *T*_1_ values of twelve model P compounds ranged from 0.61 s for phytic acid to 9.65 s for orthophosphate. In contrast, the presence of paramagnetic ion significantly shortened the *T*_1_ values of orthophosphate, pyrophosphate, and phytic acid to 1.29, 1.26, and 0.07 s, respectively, except that of deoxyribonucleic acid (DNA) remaining unchanged. Additionally, we evaluated the feasibility of improving the efficiency of quantitative ^31^P NMR analysis via addition of paramagnetic ion. Results show that, after an addition of 50 mg L^−1^ paramagnetic ions, ^31^P NMR measurement can be 3 times more efficient, attributed to the reduced *T*_1_ and the corresponding recycle delay.

## Introduction

Organic phosphorus (P) accounts for 35–65% of soil P [1], therefore the dynamics of organic P in soils plays an important role in the global biogeochemical P cycling, which is beneficial for sustainable agriculture [2–4]. Unlike soil inorganic P mainly existing as orthophosphates that can be easily identified by the classic colorimetry method [5], soil organic P that has a wide range of diverse molecular structures is difficult to analyze [6]. Therefore, advanced analytical techniques are essential to analyze the speciation and composition of organic P in the heterogeneous soils at the molecular level for comprehensive information about the P biogeochemistry in soil.

In the past 20 years, solution ^31^P nuclear magnetic resonance (NMR) spectroscopy has been developed and widely applied to speciate soil organic P after certain chemical extractions [7–9]. Because the isotropic NMR signals are well resolved and indicative for the structural information of specific P compounds, soil extracts are routinely analyzed by one-dimensional (1D) solution ^31^P NMR to quantitatively determine different organic P species [8]. Capable of extracting nearly all the bioavailable P in soil, a single-step extraction using sodium hydroxide/ethylenediaminetetraacetic acid (NaOH/EDTA) becomes the most widely used protocol for most soils and even other matrices (e.g., sediments and manures) [8, 10–13]. With the 1D solution ^31^P NMR method employed on soil extracts, in-depth knowledge about soil organic P has been obtained during the last decades, including its speciation profile in various types of soils [11–13], the biogeochemical processes of P species at various scales [14–16], and their roles in the relevant ecosystems [17, 18]. Despite the successful application in soil science, solution ^31^P NMR has major shortcomings which hamper its extensive application in this field. One main limitation is a long scanning time (~ 16 to 20 h for one sample) was required to achieve a reasonable signal to noise (S/N) ratio of NMR spectra due to the relatively low amount of P in soils, making the NMR measurement often time-limited and expensive.

A quantitative ^31^P NMR experiment on soil extracts usually needs to accumulate thousands of scans to acquire a high-quality spectrum [8]. As an important NMR experimental parameter, the recycle delay between scans that determines the whole length of the experimental time should be sufficiently long for all the ^31^P nuclear magnetization to be fully recovered back to the equilibrium state. For the 90° radio-frequency (RF) excitation pulse, the recycle delay time should be set to be five times of the spin–lattice relaxation (*T*_1_) time of the P resonances in soil extracts [19]. Otherwise, the NMR signal intensities will not be quantitative for all P species. Although *T*_1_ is the basic parameter to warrant successful NMR measurements, accurate measurements of the *T*_1_ for many soil organic P compounds have not been systematically reported.

In this study, we selected twelve model P compounds (e.g., orthophosphate, pyrophosphate, phytic acid, and deoxyribonucleic acid (DNA), etc.) to represent typical soil organic P species, and determined their *T*_1_ values using the inversion recovery method. Soil extract often contains paramagnetic ions (mainly as Fe^3+^ and Mn^2+^), which would impact the relaxation of ^31^P nuclei [19, 20]. Solution ^31^P NMR analyses were also conducted to examine the effect of paramagnetic ions on the measurement of soil organic P speciation. The objectives of this study were to determine the *T*_1_ values for several model compounds for typical soil organic P and to test the feasibility of improving the NMR analytical efficiency by paramagnetic ion addition. This research may help provide a fundamental understanding of the relaxation process of soil P, conducive to the improvement of solution ^31^P NMR analysis.

## Experimental methods

### Model P compounds

A total of twelve model P compounds were selected as the representatives of soil P. These compounds consisted of five classes, including inorganic phosphates (trisodium phosphate, tetrasodium pyrophosphate, and hexasodium tripolyphosphate), orthophosphate monoesters (sodium adenosine 5′ monophosphate, disodium d-glucose-6-phosphate, disodium β-glycerophosphate, disodium guanosine 5′ monophosphate, and phytic acid), orthophosphate diesters (sodium deoxyribonucleate Type XIV and l-α phosphatidyl choline), phosphonates (2-aminoethyl phosphonic acid), and organic polyphosphates (disodium adenosine 5′ triphosphate). All the model P compounds were purchased from Sigma-Aldrich LLC (UK).

### Pretreatment of samples for NMR analysis

All the model P compounds were prepared at both neutral and alkaline solutions to investigate the effects of the sample matrix on their spin–lattice relaxation. Each model P compound except sodium deoxyribonucleate was added to deionized H_2_O (pH ~ 7) and 1 M NaOH (pH > 13), respectively, at a total P molar concentration of 25 mM. Sodium deoxyribonucleate was added at 1 mg mL^−1^ due to its low solubility. Unless otherwise stated, 20% (v/v) D_2_O (99.9 atom % D, Sigma-Aldrich) was contained in the solution for NMR analysis.

Several samples, including trisodium phosphate, tetrasodium pyrophosphate, phytic acid, and sodium deoxyribonucleate were prepared with the addition of synthetic paramagnetic solutions at a total content of Fe and Mn of 20, 50, and 100 mg L^−1^, respectively. The paramagnetic solutions were prepared by dilution of the NaOH/EDTA extract of a certain soil sample collected in agricultural lands located in Shuangyashan, Heilongjiang Province, China (N46° 48′ 20.3″, E134° 01′ 13.9″), where grows *Glycine max*. The major physiochemical properties of the soil sample were: pH 6.90, C 56 g kg^−1^, N 13 g kg^−1^, and P 1.8 g kg^−1^. The soil sample was passed through a sieve with a 2-mm diameter mesh size and extracted by shaking 5 g of soil with 100 mL extractant containing 0.25 M NaOH and 0.05 M EDTA for 16 h at 25 °C in dark [21]. The solution sample was centrifuged at 10,000*g* for 30 min and the supernatants were filtered through 0.22-μm syringe filters. The filtrate was immediately frozen using liquid nitrogen and followed by freeze-drying. Then the lyophilized powder was redissolved in 0.25 M NaOH with a solid/solution ratio (*w*/*v*) of 1:40 to obtain the soil extract. The total P, Fe, and Mn contents of the soil extract were determined to be 101, 233, and 12 mg L^−1^, respectively, using inductively coupled plasma optical emission spectroscopy (ICP-OES, iCAP 6000, Thermo Fisher Scientific, USA). The inorganic P content (molybdate-reactive P) of the soil extract was determined to be 62 mg L^−1^ using the molybdate colorimetric method [22]. The selected P compounds were spiked into the corresponding soil extracts diluted using 0.25 M NaOH by 12.5, 5, and 2.5 times, respectively. To test the feasibility of improving the NMR analytical efficiency by paramagnetic ion addition, a synthetic soil extract sample was first analyzed. Trisodium phosphate, tetrasodium pyrophosphate, and phytic acid were simultaneously spiked into the diluted soil extract with a total Fe and Mn concentration of 50 mg L^−1^. The final P molar contents of orthophosphate, pyrophosphate, and phytic acid in the synthetic sample were about 20, 10, and 10 mM.

### Solution ^31^P NMR analysis

Solution ^31^P NMR spectra of the synthetic soil extract sample were collected using a Bruker 600 MHz solution NMR spectrometer (USA) operating at 242.98 MHz for ^31^P with a 5-mm BBO probehead at 25 °C. A 90° RF pulse (zgig pulse program), an acquisition time of 0.845 s, and a series of recycle delay times (0.1, 0.2, 0.5, 1, 2, 3.5, 14, and 50 s) were adopted with a number of scans (NS) of 128. Then, solution ^31^P NMR analysis was conducted for the undiluted soil extract and the spectra were collected using the same acquisition parameters but only with a recycle delay time of 0.1 and 2 s and a NS of 25,600. According to Cade-Menun [7], the chemical shifts of soil P compounds were classified as: phosphonates, 7 − 20 ppm; orthophosphate, ~ 6 ppm; orthophosphate monoesters, 3–6 and 6–7 ppm; orthophosphate diesters, 2.5 to− 3 ppm; pyrophosphate, ~ −5 ppm; polyphosphate, −5 to − 20 ppm. The relative abundance of each class was estimated as the relative percentage of integral area of the corresponding region to the total spectral area using the standard TopSpin software (Bruker, USA).

### *T*_1_ value measurement

The inversion recovery method was adopted for the determination of *T*_1_ value [23]. At first, the peaks of model P compounds were detected using a 90° RF pulse (zgig pulse program) and a 2 s relaxation delay with waltz decoupling. Then the *T*_1_ value for the peaks was measured using an inversion recovery pulse sequence (t1irpg program) that was a two-pulse sequence where spin populations were inverted with a 180° pulse. The recovery was monitored by using a variable delay (τ, t1 delay) followed by a 90° observation pulse. The relaxation delay was 5–40 s. The same number of scans was taken at each of the 10 τ values used until a sufficient S/N ratio was obtained. *T*_1_ values were determined by fitting the peak intensity integral in the ^31^P inversion recovery experiment with the function (1 − exp(−τ/*T*_1_), using the standard Topspin software.

## Results and discussion

### Chemical shifts of model P compounds

**Fig. 1:**
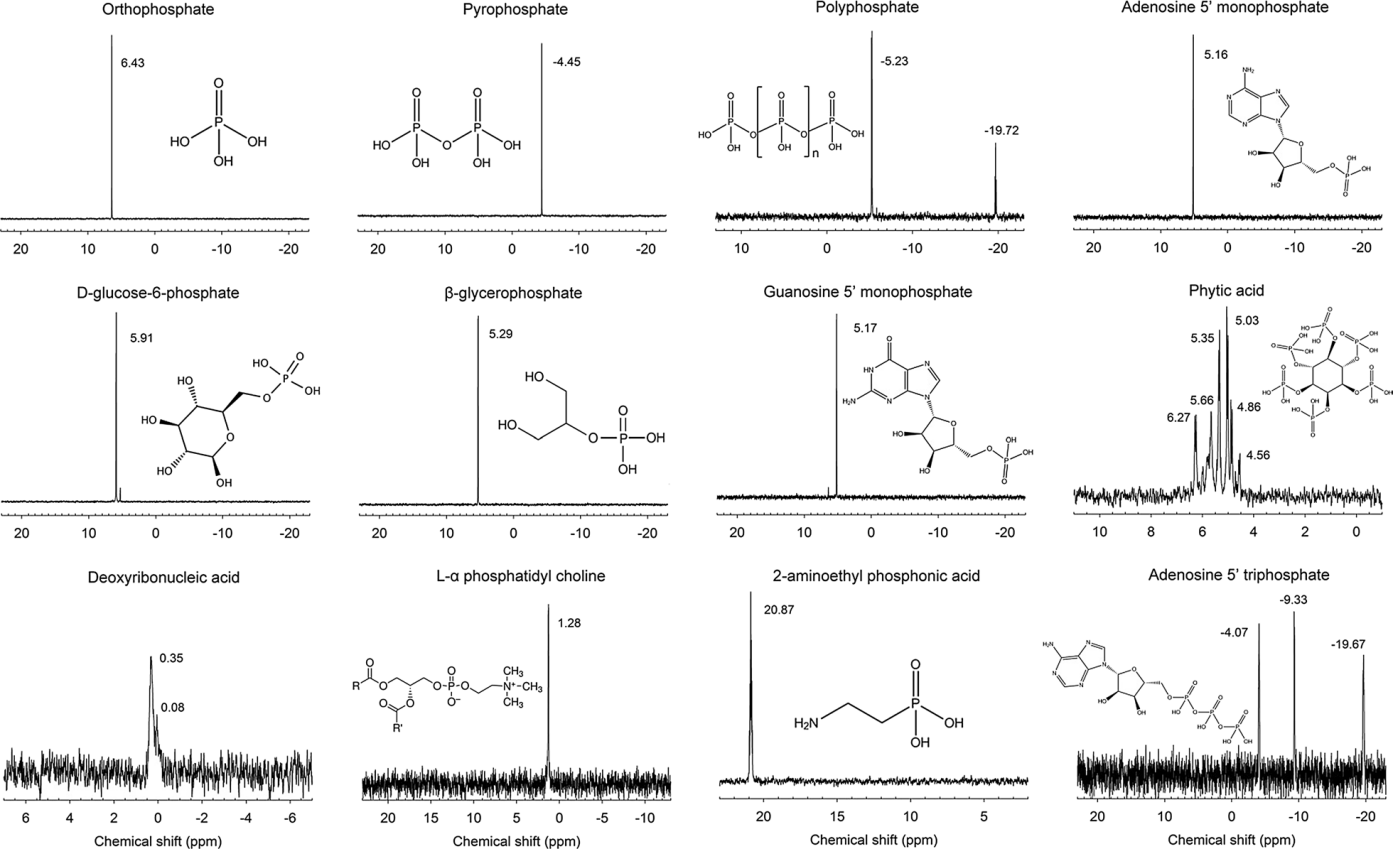
Solution ^31^P NMR spectra of model P compounds

Solution ^31^P NMR spectra of the model P compounds were shown in Fig. 1, which were all collected in alkaline condition (1 M NaOH), except that of l-α phosphatidyl choline in neutral H_2_O because it would hydrolyze in alkaline solution. The spectra of orthophosphate and pyrophosphate both showed one intense peak at a chemical shift of 6.43 and − 4.45 ppm, respectively. Two peaks were observed in the spectrum of polyphosphate (represented by tripolyphosphate) at − 5.23 and − 19.72 ppm, which were corresponding to the terminal P groups and the mid-chain P groups, respectively. Orthophosphate monoesters gave signals between 4.5 and 6.5 ppm. Unlike those of the other monoesters, the spectrum of phytic acid showed six characteristic peaks, probably resulted from the isomeric forms with different positions of the P atoms on the inositol ring in the compound used. The chemical shifts of orthophosphate diesters ranged from 0 to 1.5 ppm. The main signal of DNA was at 0.35 ppm, with a shoulder peak appearing at 0.08 ppm. The phosphonate compound resonated at 20.87 ppm, whereas the organic polyphosphate generated three characteristic peaks at − 4.07, − 9.33, and − 19.67 ppm corresponding to the three phosphates in adenosine 5′ triphosphate (ATP). The chemical shifts of all the model P compounds observed in 1 M NaOH were consistent with those reported previously in a soil NaOH–EDTA extract [6].

### *T*_1_ values of model P compounds

**Fig. 2:**
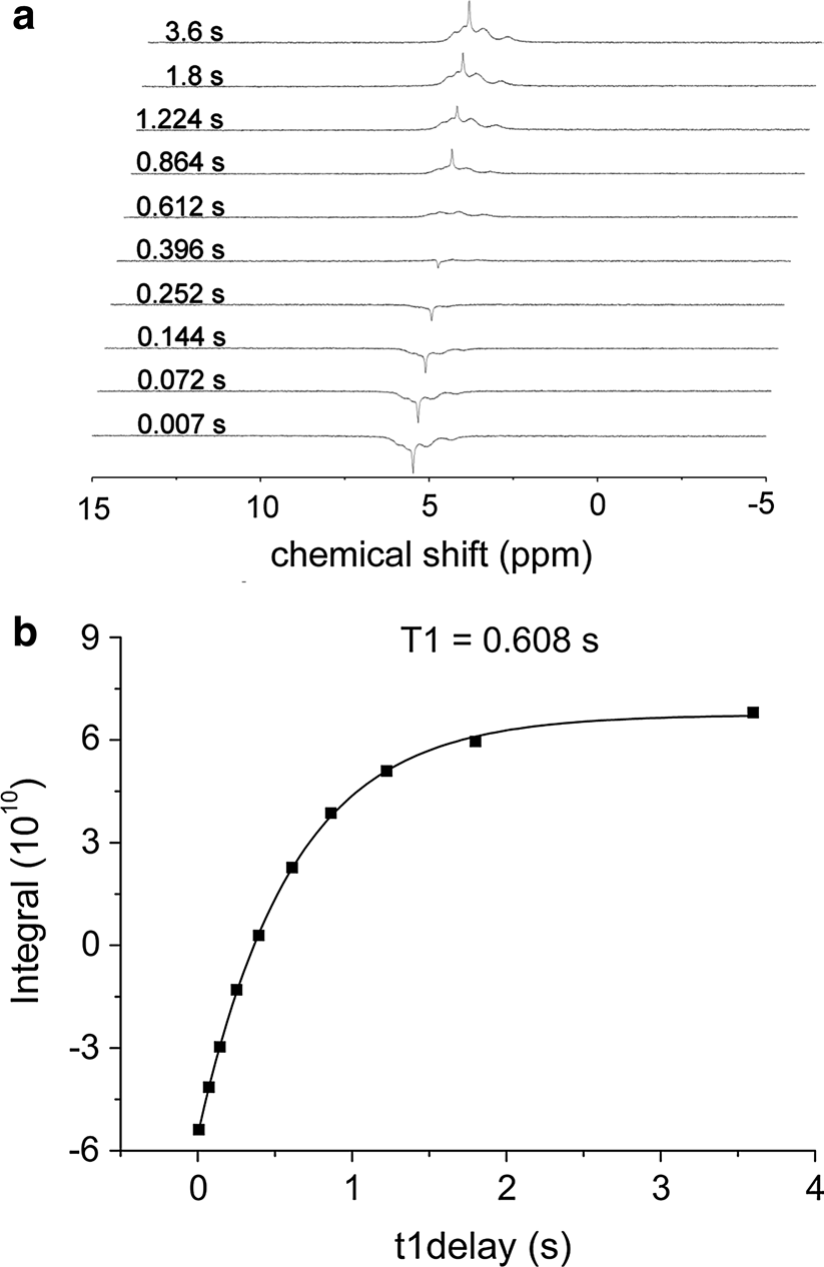
Inversion recovery method determining the *T*_1_ value of model P compounds taking phytic acid in neutral solution as an example. **a** Inversion recovery stacked spectra of phytic acid with 10 τ used and **b** regression analysis determining the *T*_1_ value of phytic acid by fitting the 10 intensity plots with the function (1 − exp(−τ/*T*_1_)

An inversion-recovery experiment was performed to determine the *T*_1_ value of phytic acid in neutral condition. The *T*_1_ value was obtained from the regression fitting to the experimental curve (Fig. 2). Results indicated that the *T*_1_ values of the model P compounds ranged from 0.61 s for phytic acid to 9.25 s for orthophosphate in neutral solution and from 0.64 s for DNA to 9.65 s for orthophosphate in alkaline solution (Table 1). For each individual P compound, an increase in pH altered the *T*_1_ value. Most of the *T*_1_ values became larger in alkaline condition, whereas those of β-glycerophosphate and 2-aminoethyl phosphonic acid were decreased. The growth of *T*_1_ value was significant for adenosine 5′ monophosphate (AMP) and ATP with a ratio of 56.1% and 73.4%, respectively. The growth ratios for the other were less than 13%, whereas the decrease ratios were less than 10%. In both neutral and alkaline conditions, dramatic differences existed in the *T*_1_ values among as well as within classes. For example, the *T*_1_ values of the phosphonate compounds were around 5 s, whereas those of the diesters and the organic polyphosphate were less than 2 s. In addition, the *T*_1_ values of d-glucose-6-phosphate were around 3 s while those of another phosphate monoester, β-glycerophosphate, were around 5 s. Besides, the *T*_1_ values of model P compounds determined without paramagnetic ions were generally longer than those in soil extracts ranging from 0.2 to 3.1 s [24]. Because natural soils usually contain 1–5% Fe in the forms of either minerals (e.g., Fe (hydr)oxides and Fe-containing clays) or organic complexes, soil extracts contain a high level of paramagnetic ions that may accelerate the spin-relaxation.

**Table 1:** *T*_1_ values of model P compounds in H_2_O (pH ~ 7) and NaOH (pH > 13)

Class	Compound	*T*_1_ (s)	
		H_2_O	NaOH
Inorganic	Orthophosphate (trisodium salt)	9.25	9.65
	Pyrophosphate (tetrasodium salt)	4.01	4.26
	Tripolyphosphate (hexasodium salt)	2.58	2.92
Monoesters	Adenosine 5′ monophosphate (sodium salt)	1.18	1.76
	d-glucose-6-phosphate (disodium salt)	3.02	3.29
	β-glycerophosphate (disodium salt)	5.16	4.92
	Guanosine 5′ monophosphate (disodium salt)	2.26	2.58
	Phytic acid	0.61	0.65
Diesters	Deoxyribonucleic acid (Type XIV, sodium salt)	NA	0.64
	l-α phosphatidyl choline	0.67	NA
Phosphonates	2-aminoethyl phosphonic acid	5.16	4.80
Organic polyphosphates	Adenosine 5′ triphosphate (disodium salt)	1.09	1.88

*NA* not applicable

### Effects of paramagnetic ions on *T*_1_ of P compounds

To investigate the *T*_1_ of soil P responding to paramagnetic ions, the *T*_1_ values of orthophosphate, pyrophosphate, phytic acid, and DNA that are ubiquitous in soils were determined in the alkaline soil extracts with different concentrations of total Fe and Mn, respectively (Table 2). Based on the *T*_1_ values of model P compounds measured with and without paramagnetic ions (Tables 1 and 2), it is obvious that solution pH only has a slight impact on the *T*_1_ value of soil P but the effects of paramagnetic ions were more significant. Although a variation about 10% were generally observed for the *T*_1_ values when solution pH was changed from neutral to alkaline (pH > 13), a decline of more than 60% would occur with a paramagnetic ion concentration of 20 mg L^−1^. Compared with those measured in the absence of paramagnetic ions, the presence of paramagnetic ions significantly reduced the *T*_1_ values of all the four P compounds. The *T*_1_ value of phytic acid was declined to about 10% of that without the addition of paramagnetic ions. The *T*_1_ values of orthophosphate and pyrophosphate were also reduced significantly after adding paramagnetic ions (100 mg L^−1^), decreased about 85 and 70%, respectively.

**Table 2:** *T*_*1*_ values of model P compounds added to soil extracts with a paramagnetic ion concentration of 20, 50, and 100 mg L^−1^

Paramagnetic ion concentration (mg L^−1^)	*T*_1_ (s)			
	Orthophosphate	Pyrophosphate	Phytic acid	Deoxyribonucleic acid
20	3.39	2.78	0.07	0.63
50	2.80	2.45	0.07	0.62
100	1.29	1.26	0.14	0.69

Furthermore, we found that the concentration of paramagnetic ion could affect the *T*_1_ of the model P compounds. For orthophosphate and pyrophosphate, the *T*_1_ value was decreased with increasing concentration of paramagnetic ions, which was consistent with the significant exponential correlation noted by McDowell et al. [19] between the *T*_1_ value of P compounds and the ratio of P concentration relative to the concentrations of Fe and Mn (i.e., P/(Fe + Mn)) in soil extracts. In contrast, the *T*_1_ value of phytic acid was declined substantially whereas that of DNA remained unchanged even with a higher concentration of paramagnetic ions (100 mg L^−1^). These differences in the responses of *T*_1_ to paramagnetic ions among soil P may originate from their relationships with paramagnetic metals at the molecular level in solution. It has been demonstrated that orthophosphate, orthophosphate monoesters, and pyrophosphate could be strongly bound to Fe and Mn [25–27], especially for phytic acid that can carry a high number of negative charges [28]. The close association between a P compound and paramagnetic ions could cause an acceleration of the relaxation process. On the contrary, orthophosphate diesters are mainly macromolecules with weak hydrophilicity (e.g., phospholipids and DNA). Their interactions with paramagnetic ions may be hampered, leading to a less significant effect on *T*_1_ value with respect to the paramagnetic ion addition. However, excessive paramagnetic ions may contribute to the formation of condensed structures for P compounds via cationic bridges enabled by sufficient trivalent ions [29, 30], thus weakening their interactions and resulting in longer *T*_1_ values (e.g., phytic acid with a total Fe and Mn concentration of 100 mg L^−1^).

### Improvement of ^31^P NMR analytical efficiency by addition of paramagnetic ion

**Fig. 3:**
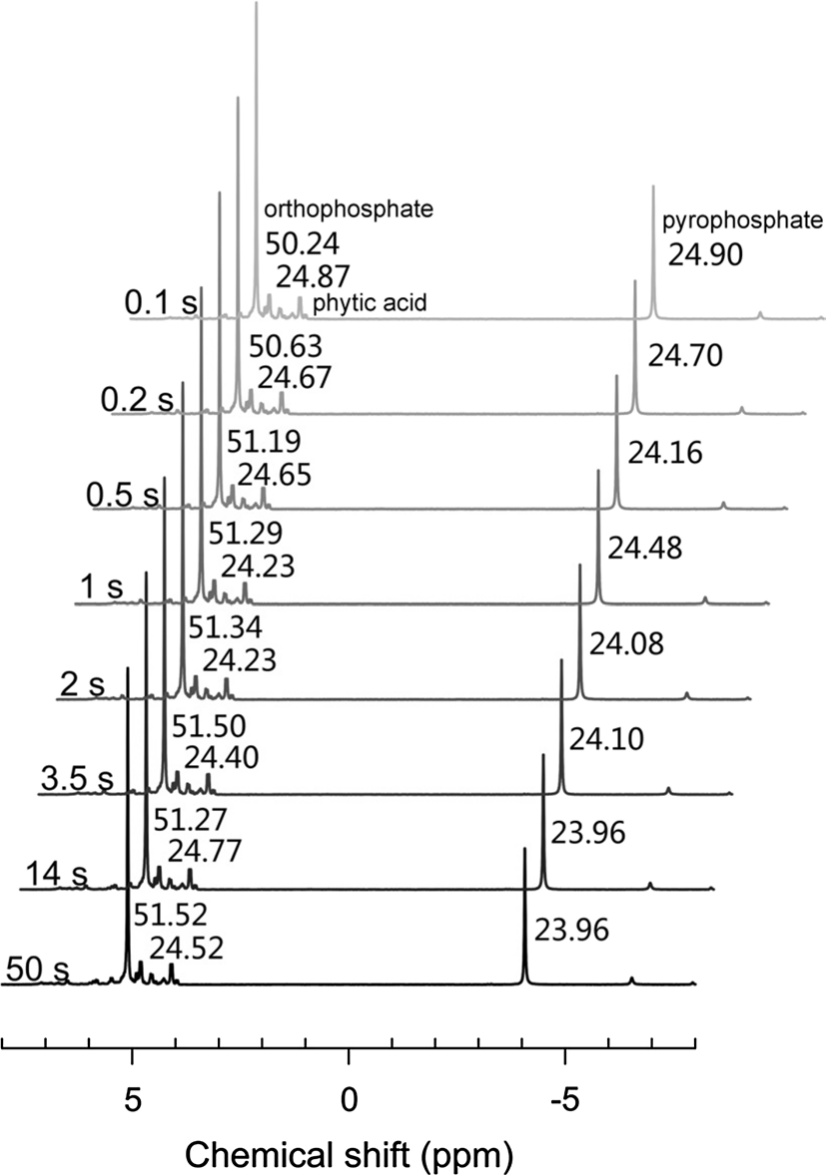
Solution ^31^P NMR spectra of the synthetic soil extract (orthophosphate, phytic acid, and pyrophosphate) with a paramagnetic ion concentration of 50 mg L^−1^ using various recycle delay times. Assignments are given for each model P compound. Numbers refer to the percentage of spectral area of each model P compound

Since the presence of paramagnetic ions could largely reduce the *T*_1_ value of various P compounds, ideally a shorter recycle delay could be applied to optimize the NMR experiments, which may save much ^31^P NMR analytical time. To test the feasibility of accelerating the NMR analysis via paramagnetic ion addition, solution ^31^P NMR spectra were collected for a synthetic soil extract containing orthophosphate, phytic acid, and pyrophosphate (P molar ratios, 2:1:1) and Fe and Mn (total content, 50 mg L^−1^) using a series of recycle delay times (Fig. 3). The relative abundances of orthophosphate, phytic acid, and pyrophosphate were 51.52, 24.52, and 23.96%, respectively, when the recycle delay time was 50 s that is long enough for a complete relaxation of all ^31^P nuclei. In comparison, when the recycle delay was shortened to 0.1 s, the relative abundances determined for the corresponding P species remained unchanged. For orthophosphate, phytic acid, and pyrophosphate species, there was only a very slight variation of 2.5, 1.4, and 3.9% observed, respectively, which suggested that a short recycle delay (i.e., 0.1 s) could be adopted if paramagnetic ion was added. Practically, the addition of paramagnetic ion can effectively reduce the analytical time without changing the accuracy of the quantitative ^31^P NMR analysis. Given that 2 s is the recycle delay time that is usually recommended for soil organic P analyses, using a 0.1 s recycle delay would decrease the duration of an NMR measurement by about 3 times in the presence of paramagnetic ions (please note that one NMR scan consists primarily of a recycle delay period and an acquisition time period (e.g., 700–800 ms) [8]).

**Fig. 4:**
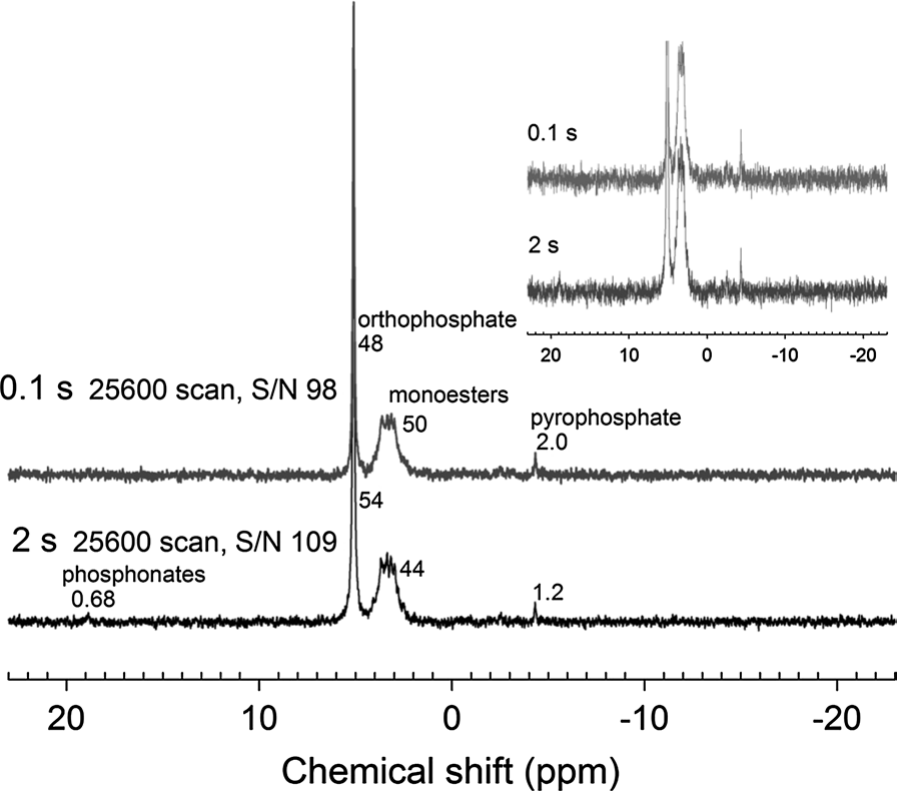
Solution ^31^P NMR spectra of the undiluted soil extract with a paramagnetic ion concentration of 245 mg L^−1^ using a recycle delay time of 0.1 and 2 s. Assignments are given for each class of P compounds above their respective peaks. Numbers refer to the percentage of spectral area for each class. The inset shows the expanded regions of phosphonate, orthophosphate monoester and pyrophosphate regions

Using solution ^31^P NMR analysis with a short recycle delay (0.1 s), we further analyzed a real soil sample (Fig. 4). With the same number of scans, we found the S/N ratio obtained of the spectrum collected using the 0.1 s recycle delay was ~ 98, which was comparable to the one collected using 2 s (~ 109). While the S/N ratio changed very little, the analytical time was apparently saved. Using the short recycle delay, it took 6.7 h for data collection whereas 20 h was needed for the long pulse delay. The *T*_1_ value of P compounds was linearly correlated with the P/(Fe + Mn) ratio in soil extracts [19]. When the total P concentration is high, it may still be necessary to add more paramagnetic ions in soil extracts to accelerate the *T*_1_ of P compounds to shorten the analytical time, unless the line broadening was increased when the spin-relaxation is too fast [31, 32]. Additionally, the interactions between P compounds and paramagnetic ions would be weakened by other substances in soil extracts, as indicated by the shorter *T*_1_ values determined in this study than those calculated through the significant correlations in soil extracts under the same P/(Fe + Mn) conditions. The use of NaOH/EDTA extracts large amounts of humic materials into the soil extract, which may chelate paramagnetic metals and hampered their effects on the *T*_1_ value [33, 34]. Therefore, although this research indicates the addition of paramagnetic ions can practically shorten the NMR experimental time and improve the analytical efficiency, further studies about the effects of complex soil matrixes on the spin-relaxation parameter in NMR spectroscopy will be still required.

## Conclusions

This study systematically determined the *T*_1_ values, a basic NMR parameter, of twelve typical soil P compounds to provide a fundamental understanding for the soil organic P analysis based on solution ^31^P NMR spectroscopy. With an addition of paramagnetic ions, the *T*_1_ values of four model P compounds ubiquitous in soil can be significantly shortened except that of DNA. Because the smaller *T*_1_ value allows for a shorter recycle delay and consequently less analytical time, the ^31^P NMR measurement for a soil extract can be about 3 times faster with insignificant loss of S/N ratio and the accuracy of the relative abundance of model P compounds. These information are critical for improving the efficiency of solution ^31^P NMR spectroscopy, but more research are still needed to evaluate whether it can be applied to all different types of soils that contain complex matrix (e.g., large molecular-weight humic substances).

## Data Availability

All data collected were reported as shown in the text and are fully available without restriction from authors upon request.

## Abbreviations

P: Phosphorus
NMR: Nuclear magnetic resonance
1D: One-dimensional
NaOH/EDTA: Sodium hydroxide/ethylenediaminetetraacetic acid
S/N: Signal to noise
*T*_1_: Spin–lattice relaxation
RF: Radio-frequency
DNA: Deoxyribonucleic acid
ICP-OES: Inductively coupled plasma optical emission spectroscopy
NS: Number of scans
ATP: Adenosine 5′ triphosphate
AMP: Adenosine 5′ monophosphate
P/(Fe + Mn): Ratio of P concentration relative to the concentrations of Fe and Mn
